# Nanotag luminescent fingerprint anti-counterfeiting technology

**DOI:** 10.1186/1556-276X-7-262

**Published:** 2012-05-22

**Authors:** Stefan Johansen, Michal Radziwon, Luciana Tavares, Horst-Günter Rubahn

**Affiliations:** 1 Nano SYD, Mads Clausen Institute, University of Southern Denmark, Alsion 2, Sønderborg, DK6400, Denmark

**Keywords:** Anti-counterfeit, Organic nanofibres, Molecular beam epitaxy, Process optimisation

## Abstract

We describe a method to fabricate, transfer and validate via image processing nanofibre-based, unique security marks (‘nanotags’) for anti-counterfeiting purposes. Epitaxial surface growth of oligophenylenes on a heated muscovite mica crystal results in a thin film of mutually aligned nanofibres with dimensions of tens of nanometres in height, hundreds of nanometres in width and tens to hundreds of micrometres in length. By applying a shadow mask, a film pattern is generated which contains only sparse, randomly grown nanofibres, which in turn represent a unique ‘fingerprint’ of the growth area. This fingerprint can be transferred on an adhesive tape as a label of a product, imaged using low magnification microscopy, digitalised and stored in a database. Infrared surface heating, enforced cooling and load lock transfer makes the fabrication process fast and scalable to mass production.

## Background

Counterfeiting is a steady and increasingly more important problem in all kind of commercialised production. Counterfeiting does not only inherently violate intellectual property rights of inventors and producers, but it also induces a real danger potential to customers, e.g. in the case of falsified drugs. A usual approach to anti-counterfeiting is to apply unique markers to the product in question. Unfortunately, readily available, advanced printing technologies provide simple access to high quality counterfeiting of such markers, especially those which are based on holographic patterns [1]. Given this background, it is not surprising that a solution to this problem has to base on the introduction of new technologies which are more difficult to counterfeit. Bottom up nanotechnology is one of these more recent approaches, which can help addressing the problem since it *per se* can imply a stochastic generation of unique and irreproducible patterns of nanoscaled objects.

The principal problem with the use of bottom-up nanotechnology for the generation of unique anti-counterfeiting markers is the inherent optical invisibility of objects that are smaller than half the wavelength of the imaging light. Hence, the uniqueness of nanomarkers created by tailored nanoscaled objects becomes obvious only if one either employs state-of-the-art imaging technology (e.g. scanning electron or atomic force microscopy) or if one bases the detection on second-order effects, e.g. unique optical appearance as for quantum dots [2]. For the purpose of anti-counterfeiting, it would, of course, be most interesting to have markers; the uniqueness of which can be detected on various identification levels, from bare eye over simple to advanced instrumentation.

A way to overcome this problem is to implement nanoscaled objects that possess a macroscopic dimension, e.g. 1D nanofibres, instead of quantum dots. In this work, we use luminescent markers generated by surface-grown patterns of organic nanofibres [3]: these nanofibres exhibit bright, polarised and spatially strong anisotropic luminescence upon UV excitation [4] or current injection (L Tavares, J Kjelstrup-Hansen, and HG Rubahn, unpublished work), are visible by the naked eye since one of their dimensions is macroscopic and show unique patterns of mutually parallel-oriented entities under low magnification microscopy, which allows simple identification of individual tags made of these fibre arrays (HG Rubahn, S Johansen, and M Radziwon, unpublished work). The fibres are also chemically inert and withstand temperatures of more than 100°C. Ambient light does not cause photo-oxidation (‘bleaching’) of the nanofibres. However, strong UV excitation causes photobleaching and eventually destroys the fibres, but this process can be avoided by appropriate surface coating [5].

However, since the ordered molecular crystals from which these nanofibres are built are only weakly van der Waals bound; they are mechanically instable if released from the growth substrate, and thus, further manipulation (transfer [6] and integration) becomes extraordinarily difficult. A further challenge is large-scale production of nanofibre-based anti-counterfeit tags since the epitaxial growth state-of-the-art process of these nanoaggregates takes a significant amount of time - usually several hours.

In this present paper, we discuss results that show how to optimise organic nanofibre growth technology towards fast production (minutes rather than hours), how to make unique patterns easily identifiable using a shadow masking technology and how to integrate them into cheap and unique nanotags on adhesives.

## Methods

**Figure 1:**
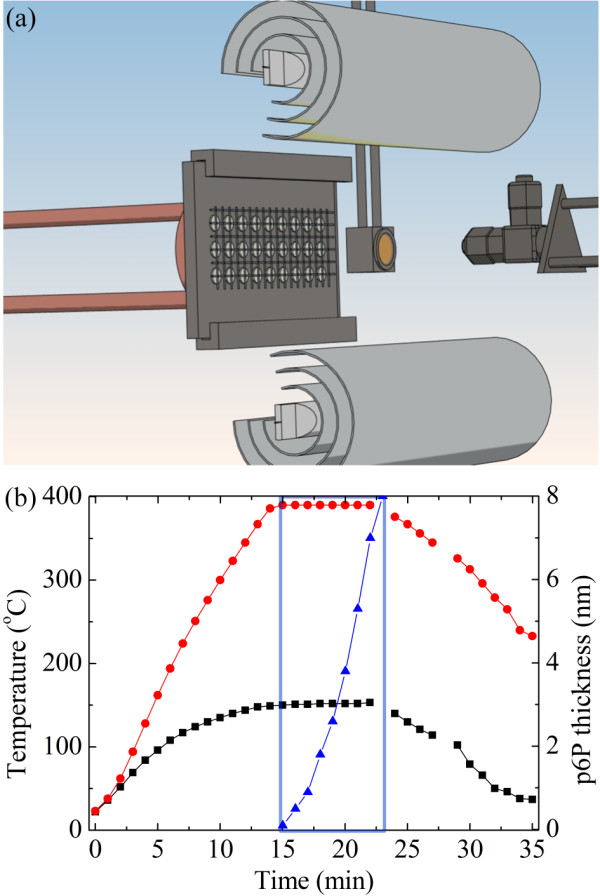
**Fabrication process.** (**a**) Sketch of the vacuum apparatus for growing nanofibres. The Knudsen cell with the p6P molecules is situated on the right side; the cooled sample holder with mica discs and masks is on the left side. The quartz microbalance is sitting to the right from the sample holder. Above and below the sample holder are the infrared heating bulbs with heat shields. (**b**) Deposition process timeline for the growth of p6P nanofibres that constitute a nanotag. Red dots, temperature of the p6P oven; black squares, temperature of the mica substrate; blue triangles, nominal p6P film thickness.

The nanofibre tags are fabricated by molecular beam epitaxy of para-hexaphenylene (p6P) molecules from a heated (663 K) Knudsen cell onto a freshly cleaved muscovite mica disc, which is brought during deposition to a surface temperature of 423 K via infrared heater placed inside the vacuum apparatus. Figure 1a shows a sketch of the experimental deposition apparatus with the process timeline in Figure 1b that is denoting the temporal evolution of Knudsen cell temperature, mica disc surface temperature and deposited film thickness. The time slot during which organic nanofibres are grown is also denoted in Figure 1b. With the present setup, the time necessary for the production of a nanotag batch is less than 0.5 h.

**Figure 2:**
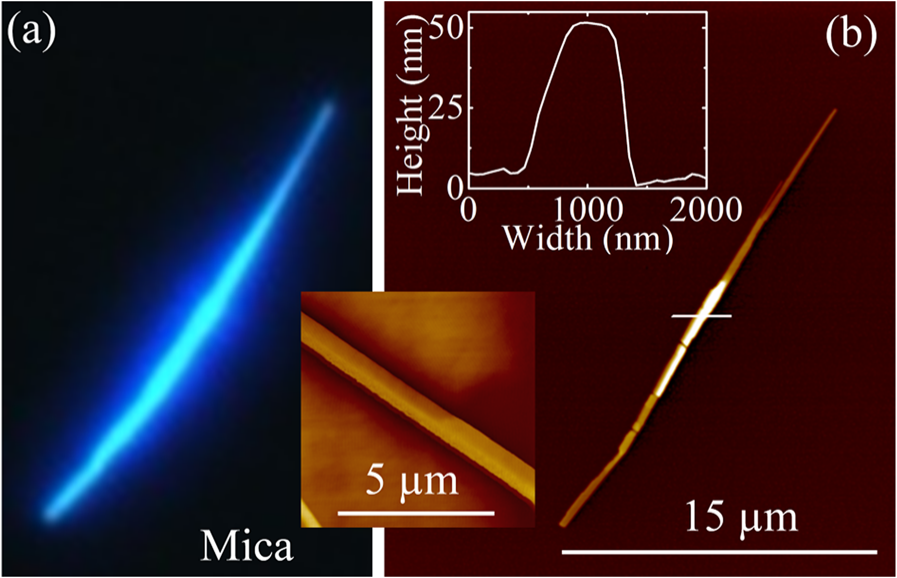
**Microscopic images of p6P nanofibre.** (**a**) Epifluorescence image (365-nm UV excitation) of an isolated p6P nanofibre. (**b**) AFM image of the same fibre, denoting typical dimensions: width, 550 nm; height, 50 nm; and length, 25 μm. Inset: AFM image of an individual fibre. Note the breaks along the fibres' long axis as well as the characteristic step edges, which indicate the crystalline nature of the material.

Epifluorescence and atomic force microscopy (AFM) images of an exemplary as grown p6P nanofibre on mica is shown in Figure 2a,b. The fibre has the following dimensions: width, 550 nm; height, 50 nm; and length, 25 μm. It is formed from p6P molecules that are grown parallel to the substrate plane in a herringbone order with the long axes of the molecules nearly perpendicular to the long axis of the crystalline fibre [4]. The fibre is thus essentially a p6P molecular crystal as indicated in the AFM image by characteristic facets on the fibre surface (see inset in Figure 2). The orientation of the molecules results in strong dichroism, i.e. polarised emission and absorption properties. The blue light emitted following UV (365 nm) excitation is spatially anisotropic [7] and consists of well-defined vibronic bands with virtually no defect emission [8].

The overall shape of the nanofibre tag as fabricated via vacuum deposition is governed by a round mask in front of the mica disc. The mask has a further shadowing cross in the middle, made of 300- to 700-mm-thick metal wires. On the mica substrate surface, the fibres form a densely packed layer of uniform thickness, which can be changed by varying the deposition rate and exposure time of the molecular beam. Due to the bright luminescence of p6P thin films, a mass thickness of 8 nm is sufficient for easy visibility of the fibre tag under LED UV illumination. The mass thickness is controlled by a quartz microbalance. In the shadowed area, the density of fibre growth is strongly decreased. However, fibres that started growing in the dense area extend into the shadowed regime, thus forming, via random growth, a unique nanofibre pattern.

Next, the as-grown fibres on the mica disc are covered by a transparent adhesive tape, which not only does not significantly affect the blue emission of the fibres, but also serves as a protective cover. By peeling off the tape from the substrate, the mica disc supporting the fibres is cleaved to an ultrathin and transparent layer. Hence, the fibres on the ultrathin mica disc are surrounded by an adhesive area and can be directly mounted on the product to be marked. By shaping the shadow mask in a unidirectional manner, a fiducial point on the surface of the tag can always be identified in a low magnification microscopic image of the surface; the unique nanofibre pattern can thus be detected and extracted by an image processing software.

## Results and discussion

**Figure 3:**
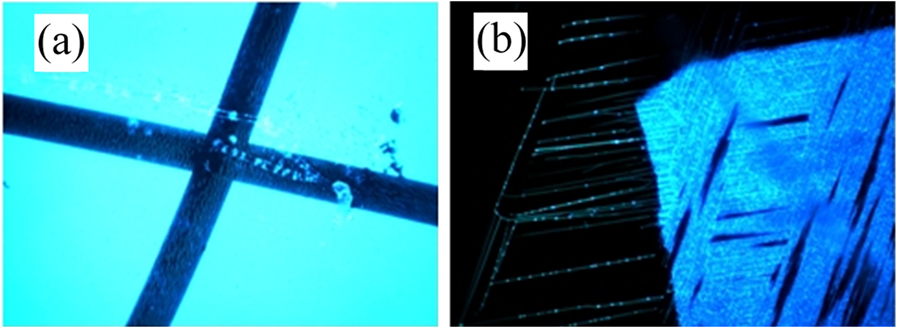
**Microphotograph of nanofibre tag.** (**a**) Epifluorescence image (excitation, 365 nm; magnification, ×10) of shadow-masked array of organic nanofibres. Distinct fibres are seen in the shadowed areas while non-shadowed consists of structureless film. (**b**) Blow-up (magnification, ×50) of a corner of the fibre-covered square, showing that individual fibres are grown underneath the masked area.

In Figure 3, an epifluorescence image of a shadow-masked array of organic nanofibres is shown. In Figure 3a, the fibre array has been illuminated with 365-nm UV light and an objective magnification of × 10. In Figure 3b, a blow-up of a corner of the fibre-covered square reveals that the quasi-continuously luminescent area consists of a dense nearly structureless film, while individual parallel-oriented nanofibres are revealed in the shadowed area. We note that illumination with a simple commercial ‘moneychecker’ (385-nm low-power UV diode) results in a similar image (not shown) since the absorption band of p6P below 400 nm is quasi-continuous. Applying an analyser in front of the CCD camera showed that the blue light, which is centred around 425 nm, is strongly polarised.

It is well known that p6P nanofibre growth on mica occurs in several steps: first, a wetting layer of molecules is formed on the substrate, followed by the formation of small clusters. Due to the surface temperature, these clusters are mobile and form aggregates. Since p6P is a rod-like molecule, the aggregates grow via side-to-side attachment of molecules, which limits the growth to one dimension on the surface. Due to the mobility of molecules and clusters on the surface of the aggregates, growth normal to the surface is also hindered. As a result, fibre-like structures with well-defined molecular order are formed. The orientation of the fibres is given by the orientation of the molecules, and this in turn is dictated by growth epitaxy [9]. Due to this characteristic growth process, the mica-grown nanofibres are always mutually aligned, but the individual pattern is not reproducible.

**Figure 4:**
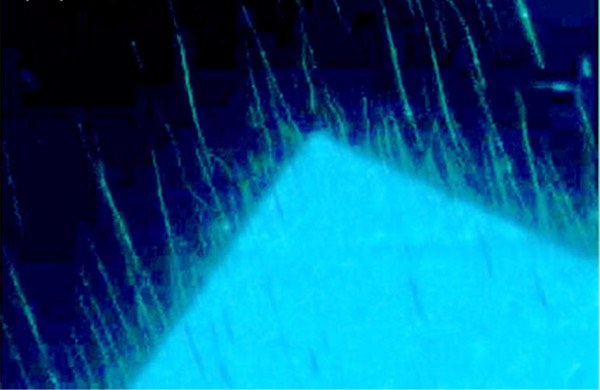
**Digitally magnified corner of one of the fibre-covered squares.** It shows fibres extending from the square into the shadowed areas.

After growth, the nanofibres are covered with a transparent adhesive film. The epifluorescence image is collected from the nanofibres through the tape with a 2.2-Mpixel camera, which is used to inspect a nanotag that has been transferred onto a product to be labelled. If one digitally magnifies a corner of one of the fibre-covered squares, fibres become easily visible. Figure 4 shows the nanofibres through the transparent adhesive under low magnification microscopy. Furthermore, it protects the fibre arrays against mechanical damage. Alteration of the nanofibre growth pattern is not observed after transfer and UV imaging.

**Figure 5:**
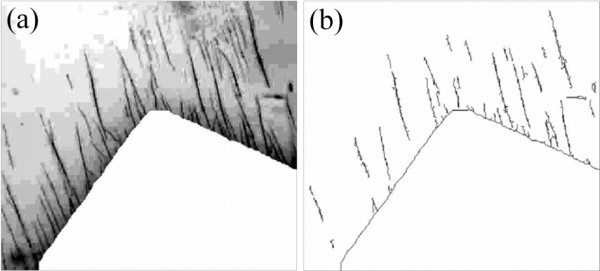
**Image processing results.** (**a**) Contrast-enhanced greyscale image of Figure 4 with non-shadowed domain removed. (**b**) Binary output of image processing.

In what follows, we will concentrate on the extracted area of the image. Figure 4 shows the selected region of interest; the image processing chain starts with colour filtering and conversion of the image into greyscale. By use of morphological operations and image arithmetic, the non-shadowed domain with dense fibres is removed from the image, while the remaining features are enhanced (see Figure 5a). Next, the image is transformed into binary format, and usage of morphological thinning results in the image in Figure 5b.

The processed image consists of two types of lines: baselines being the borders of the masked region and lines corresponding to fibres which have grown underneath the mask. The angles at which those fibres grow, and especially the coordinates of their terminators, are stochastic parameters. Therefore, this information derived from the image is truly unique for each sample.

**Figure 6:**
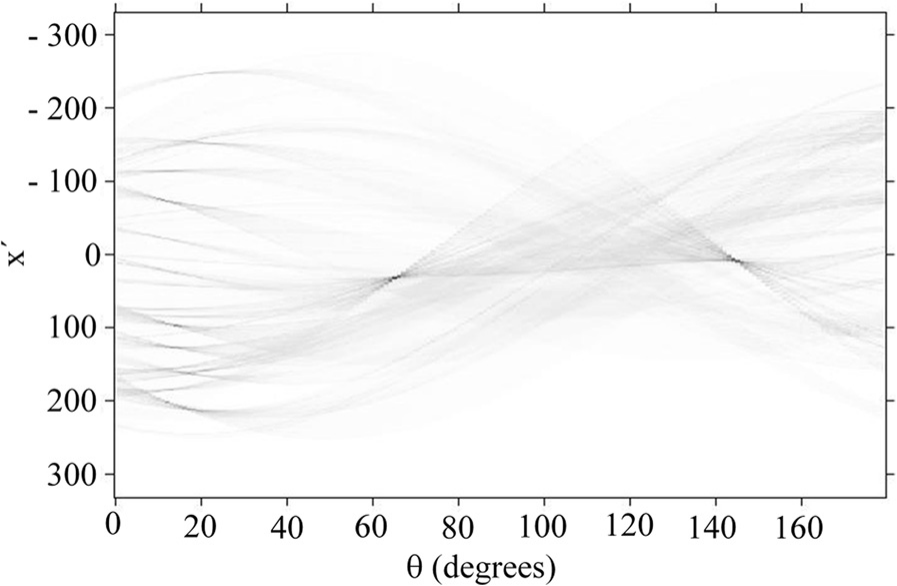
**Sinogram of Figure**5b**.** Major maxima (at [63°, −40] and [145°, −35]) correspond to the border lines of the non-shadowed domain. The maxima at abscissas around 17° indicate positions and angles of characteristic nanofibres.

The tool which is used for deriving those data is the Radon transform. It is the integral transform, which integrates the image over straight lines with baseline inclination and bin position as parameters. The transform results in a (*m* × *n*) sized matrix, where *m* is the number of bins on the *x* axis and *n* is the number of angles at which the image is being analysed. A sinogram (output of Radon transform) of our output image is shown in Figure 6. Here, the position of each of the local maxima represents a detected line. One can easily identify two major maxima (at 63°, −40; at 145°, −35), which correspond to the border lines. The maxima at abscissas around 17° indicate distinct nanofibres. The fact that the angle values are nearly the same for all fibres indicates mutual alignment of the fibres. Further processing includes selection of an arbitrary number of *N* highest maxima which correspond to the longest fibres. The relative angles between the long axes of the detected fibres and the bisection of the borderlines, together with the distance of the lines coinciding with the fibres' long axes from the shadow mask corner, form a unique rotation invariant coordinate matrix. This coordinate matrix is used as a digital fingerprint.

Distinction between two coordinate matrices of different samples relies therefore on the ability to position the fibre in relation to the border of the masked region. Due to the implemented image processing sequence, the fibre needs to have at least a 4-pixel width to be detected, and the minimum distance between fibres is 8 pixels. This limits the average number of detectable fibre terminations on the borderline to √2/8 × the image width which, for exemplary images, equals to 110. If we want to detect only the 10 most distinct fibres, it gives over 4 × 10^13^ combinations (10 out of 110) of fibre starting points solely. In addition, this number will increase by a factor of about 10^3^ due to variations in the fibre tilt angles. Therefore, the parameter variation is large enough for most counterfeit applications.

**Figure 7:**
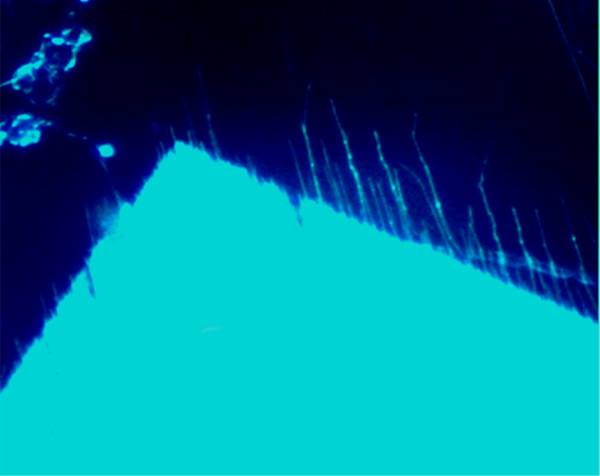
Extracted and digitally magnified ROI of × 20 magnification image of another sample obtained under UV illumination.

**Figure 8:**
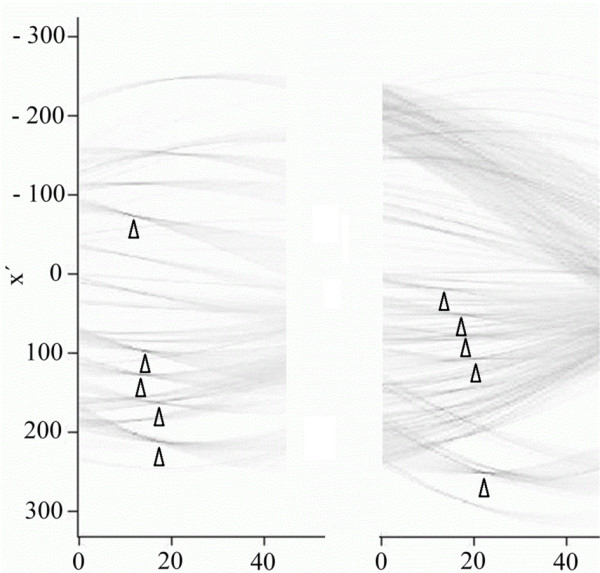
**Comparison of sinograms of Figures**4**and**7**.** Triangles indicate detected coordinates of five most distinctive fibres.

**Figure 9:**
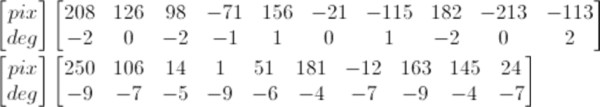
**Coordinate matrices extracted from sample 1 (Figure**4**) and sample 2 (Figure**7**), respectively.** First row represents the distance of a line coinciding with detected fibre from the tip of the mask (±4 pixel), while the second is the tilt angle with respect to mask shadow bisection line (±1°).

Examples of two distinct samples are shown in Figures 4 and 7; their respective sinograms are in Figure 8, and their extracted coordinate matrices are in Figure 9. As can be seen, those matrices are clearly distinguishable.

After fabrication and UV-assisted optical inspection of each produced marker, its coordinate matrix is stored in a secured database, paired with information on the particular tag (serial number, production date etc.). When a product is marked with a tag, it can always be determined whether it is genuine by taking a low magnification UV-exposed microscopic photograph of the tag and extracting the fingerprint data to compare it with that already existing in the database.

## Conclusions

In this article, we have presented a method to generate unique nanotags for product anti-counterfeiting using bottom-up grown arrays of ordered organic nanofibres. The resulting nanotags can be readily transferred onto arbitrary products using transparent adhesive foils. Due to the simplicity of fibre array generation, there is no principal obstacle for upscaling the method to large-scale production.

## Abbreviations

AFM: atomic force microscopy; p6P: para-hexaphenylene.

## Competing interests

The authors declare that they have no competing interests.

## Authors’ contributions

SJ built the vacuum apparatus, was involved in the preparation of the nanofibres and tags, and helped revise the manuscript. MR was involved in the image processing, interpretation of data and revision of the manuscript. LT was involved in the growth, integration and characterisation of the nanofibres and tags, and also revised the manuscript. H-GR helped to analyse the data and wrote the manuscript. All authors read and approved the final manuscript.
